# Dunglison’s Dictionary of Medical Science, Twenty-first Edition, with Appendix

**Published:** 1895-12

**Authors:** 


DUNGLISON'S DICTIONARY OF MEDICAL SCIENCE, TWENTY-FIRST EDITION, WITH APPENDIX.—Containing a full explanation of the various subjects and terms of Anatomy, Physiology, Medical Chemistry, Pharmacy, Pharmacology, Therapeutics, Medicine, Hygiene, Dietetics, Pathology, Surgery, Ophthalmology, Otology, Laryngology, Dermatology, Gynecology, Obstetrics, Pediatrics, Medical Jurisprudence and Dentistry, etc., etc. By Robley Dunglison, M. D., LL. D., late Professor of Institutes of Medicine in the Jefferson Medical College of Philadelphia. Edited by Richard J. Dunglison, A. M., M. D. New (21st) edition, thoroughly revised, greatly enlarged and improved, with the Pronunciation, Accentuation, and Derivation of the terms. In one magnificent imperial octavo volume of 1225 pages. Cloth, $7.00; leather, $8.00. Thumb-letter Index for quick use, 75 cents extra. Lea Brothers & Co., Publishers, Philadelphia. 1895.

Our readers will remember that a review of the 21st edition of "Dunglison's Dictionary of Medical Science," appeared in our columns some months since. We are now in receipt of an appendix to this great medical lexicon. This "appendix" contains twenty-four pages, which makes the book more complete and brings it up to the present date. The price of the book, including the "Appendix," will remain the same as heretofore. H.

